# Preparation, Optimization, and Stability of Isosulfan Blue-Loaded Human Serum Albumin-Structured Nanocolloids

**DOI:** 10.1021/acsomega.5c02567

**Published:** 2025-07-03

**Authors:** Emre Ozgenc, Meliha Ekinci, Evren Gundogdu, Makbule Asikoglu, Derya Ilem-Ozdemir

**Affiliations:** 37509 Ege University, Faculty of Pharmacy Department of Radiopharmacy, Bornova, Izmir 35040, Turkey

## Abstract

In an attempt to develop nanoparticles for drug delivery systems, human serum albumin (HSA), a protein carrier for drugs, is a suitable choice. HSA nanoparticles have been widely used in drug delivery systems and have applications in various diseases. The administration of HSA nanoparticles, particle size, surface charge, and drug loading are important parameters to consider. The present study aimed to develop HSA-based nanoparticles containing isosulfan blue (ISB) dye by using a nanoprecipitation procedure. The developed nanoparticles were characterized by scanning electron microscopy and dynamic light scattering (DLS). The effects of factors such as solvent/nonsolvent ratio, pH, cross-linker, dye, and surfactant concentration on the physicochemical parameters of the formulations were optimized. Also, in vitro stability studies of nanoparticles were measured in different conditions for up to 3 months. According to the results obtained, the spherical nanoparticles had particle sizes of 93.88 ± 1.86 and 113.90 ± 1.91 nm, PdI values of 0.19 ± 0.01 and 0.44 ± 0.02, and negative charges of −26.0 ± 2.87 and 57.4 ± 3.86 mV. The loading amount of ISB for ISB-HSA nanoparticles was 15–20%. Also, nanoparticle formulations were stable under different conditions and did not exhibit a substantial change in DLS results. Current sentinel lymph node tracers, like free ISB, suffer from rapid clearance (<2 h) and photodegradation. This study develops stable HSA nanocolloids to overcome these limitations. In conclusion, ISB dye-loaded HSA nanoparticles in stable nanocolloidal structures with ideal properties were prepared.

## Graphical abstract

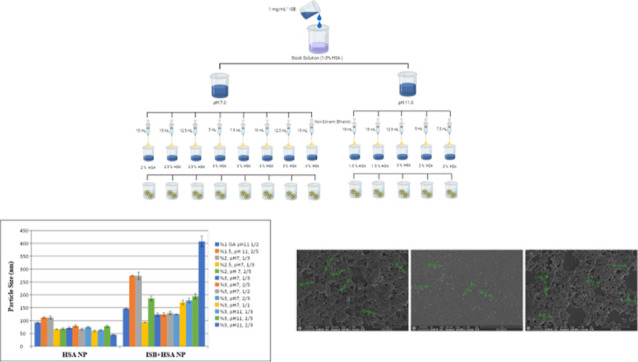

## Introduction

Nanoparticle-based drug delivery systems have been extensively investigated as a robust strategy in targeted drug delivery in pharmaceuticals.^1–3^ Human serum albumin (HSA), the most prevalent protein in human blood, is a natural and suitable substrate for the production of drug delivery nanoparticles. Albumin nanoparticles have attracted much attention in recent years due to their remarkable capacity to load a variety of drugs nonspecifically and their safety when administered in vivo.^4,5^ Albumin-bound nanoparticles can promote tumor drug accumulation using the well-known passive targeting mechanism, enhanced permeability, and retention (EPR) effect. According to the EPR effect, a tumor’s blood vessel walls are dilated, leaky, or damaged, and the endothelium is disordered with fenestrations that are only a few nanometers wide.^6,7^ Nanocarriers between 20 and 250 nm can extravasate fluid from the vessels into the interstitial fluid. Nanoparticles can pass through endothelial holes with diameters between 10 and 1000 nm. Additionally, a tumor’s lymphatic veins are not functioning correctly, contributing to the ineffective drainage of fluids from the tumor tissue. Nanocarriers that enter the tumor are not effectively removed; thus, they accumulate.^8^

Numerous physicochemical techniques, such as thermal gelation,^9–11^ emulsification,^12–14^ desolvation (coacervation),^15–17^ and nanoprecipitation,^18,19^ have been proposed to synthesize albumin-based nanocarriers. Because of their simplicity and reproducibility, the nanoprecipitation method seems to be the most suitable. The newly formed albumin nanoparticles are unstable; hence, an additional step of stabilization or cross-linkage must be carried out to increase their half-life in an aqueous environment and/or prevent the development of protein macroaggregates.^20,21^ In general, one of the methods used most to stabilize albumin nanoparticles is cross-linkage with glutaraldehyde. Glutaraldehyde is quite helpful for this purpose.^22,23^

Isosulfan blue (ISB) is the first dye to detect sentinel lymph nodes in breast cancer. After injection, it is transported to the local proteins, especially bound to albumin.^24^ The most essential characteristics of HSA nanoparticles are particle size, shape, and zeta potential.^25,26^ Therefore, controlled experiments were designed to optimize HSA nanoparticles.^27^

This study aimed to develop and characterize HSA nanoparticles as drug carriers. For this aim, HSA nanoparticles were prepared using the nanoprecipitation technique. The developed formulations were characterized by assessing particle size, zeta potential, and the morphology profile. The effects of factors such as solvent/nonsolvent ratio, pH, cross-linker, dye, and surfactant concentration on the physicochemical parameters of the formulations were optimized. Also, in vitro stability studies of nanoparticles were measured in different conditions for up to 3 months.

## Results and Discussion

A detailed preformulation study was carried out to prepare nanocolloidal formulation of ISB-loaded HSA nanoparticles. A series of controlled experiments investigated the effect of many factors on the formulation’s physicochemical properties.

### Nonsolvent Volume Effect

Figure 1 shows the change in particle sizes of the formulations (pH: 7.0) prepared using different solvent/nonsolvent volume ratios. The study determined that the solvent/nonsolvent volume ratio is the critical parameter for the formulation, but the main factor affecting particle size was HSA concentration.

**Figure 1:**
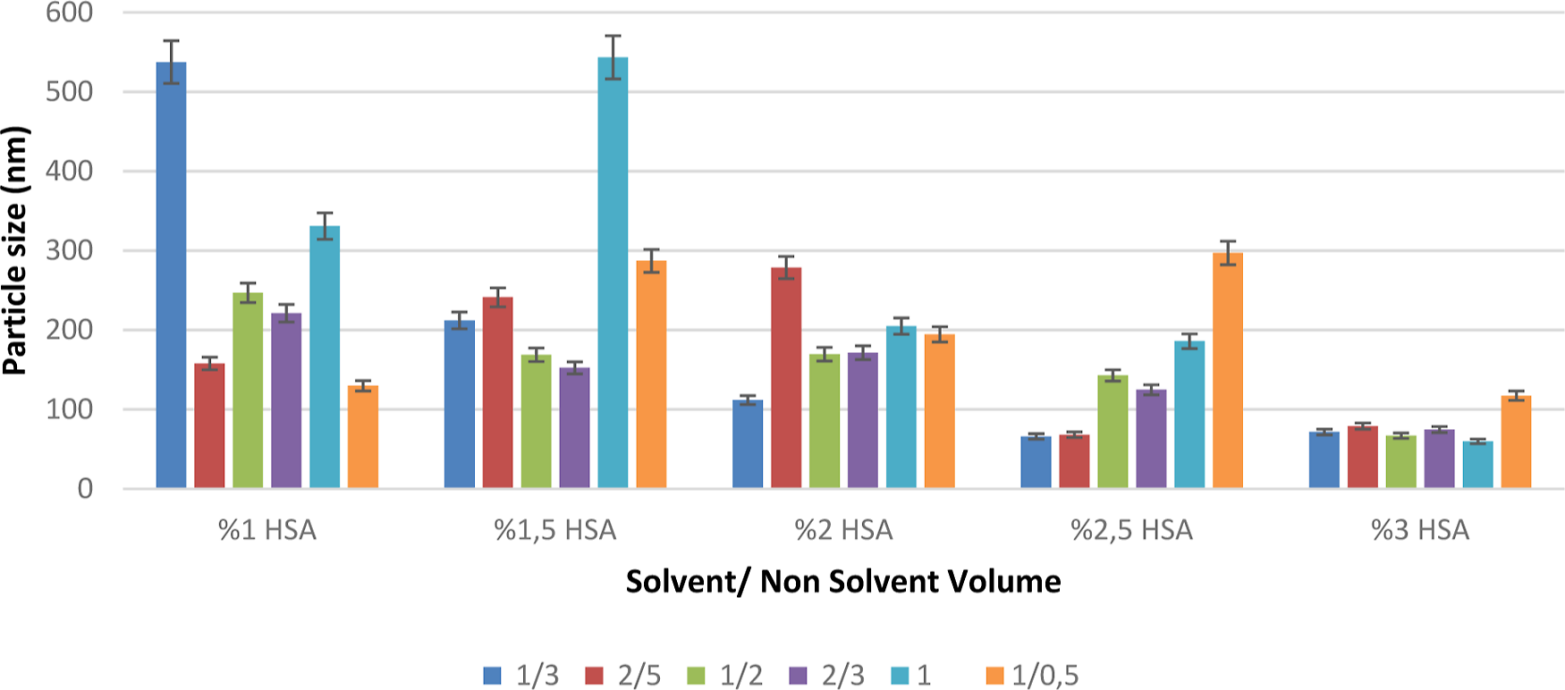
Effect of solvent/nonsolvent volume ratio on the particle size.

### pH Effect

The particle sizes of formulations prepared using different solvent/nonsolvent volume ratios with each concentration of HSA prepared at pH 7.0 and 11.0 were analyzed. The results are presented in Figure 2a–e.

**Figure 2:**
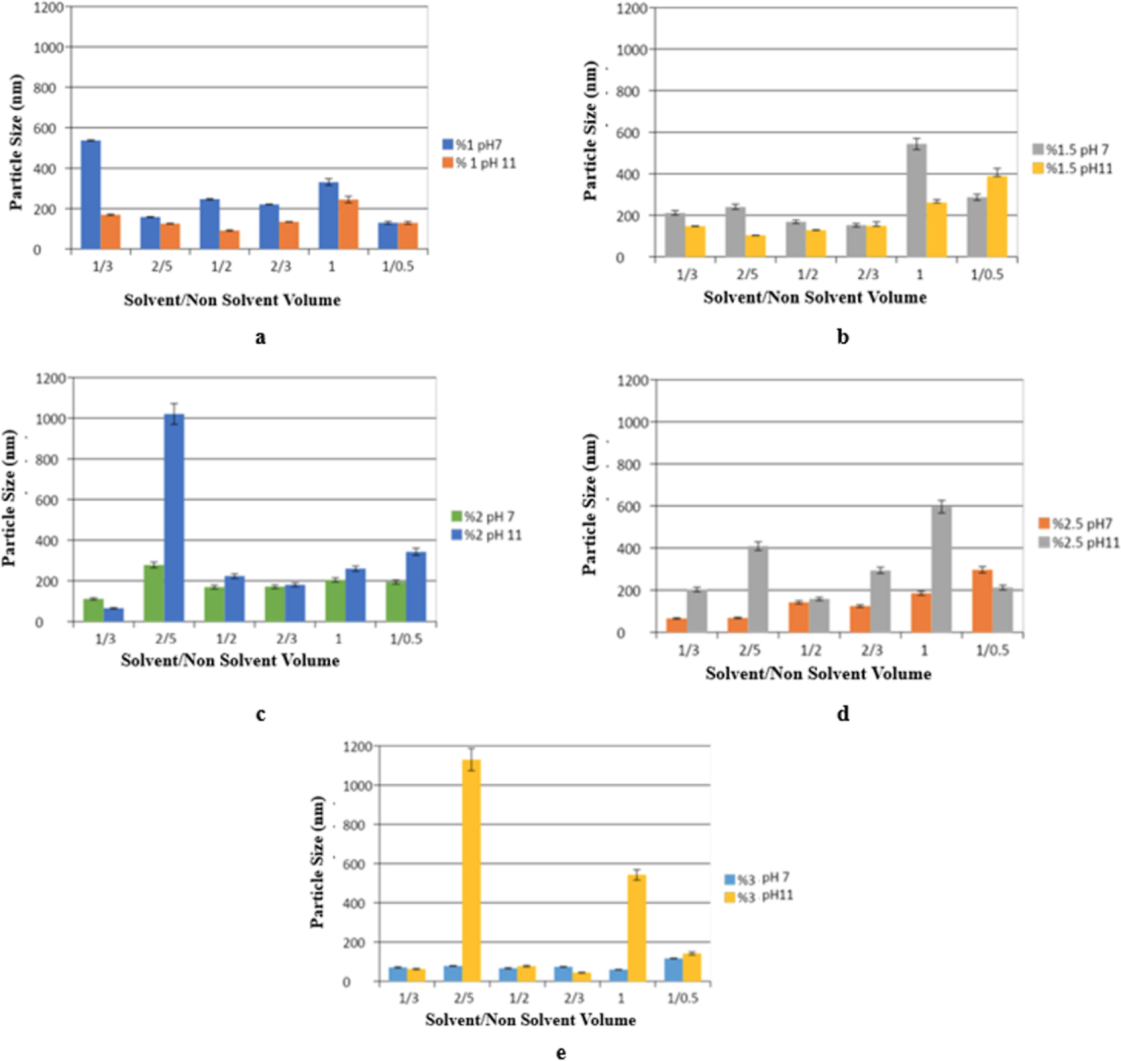
Particle sizes of formulations containing different solvent/nonsolvent volumes (1/3, 2/5, 1/2, 2/3, 1, and 1/0.5) and prepared from stock at various ratios: (a) 1%, (b) 1.5%, (c) 2%, (d) 2.5%, and (e) 3% HSA at pH 7 and 11.

The results concluded that it is incorrect to make a standard generalization based on the pH change alone and that evaluating each formulation within itself would be appropriate. From here on, further studies were continued with formulations with particle sizes around 100 nm and below. The contents and codes of the formulations studied are presented in Table 1.

**Table 1:** Contents and Codes of the Formulations with Particle Size ≤100 nm

formulation	HSA (%)	pH	solvent/nonsolvent
F1	1.0	11.0	1/2
F2	1.5	11.0	2/5
F3	2.0	7.0	1/3
F4	2.5	7.0	1/3
F5	2.5	7.0	2/5
F6	3.0	7.0	1/3
F7	3.0	7.0	2/5
F8	3.0	7.0	1/2
F9	3.0	7.0	2/3
F10	3.0	7.0	1/1
F11	3.0	11.0	1/3
F12	3.0	11.0	1/2
F13	3.0	11.0	2/3

### Dye (ISB) Concentration Effect

Figure 3 shows the particle size, zeta potential, and PDI values of the formulations containing different ISB concentrations.

**Figure 3:**
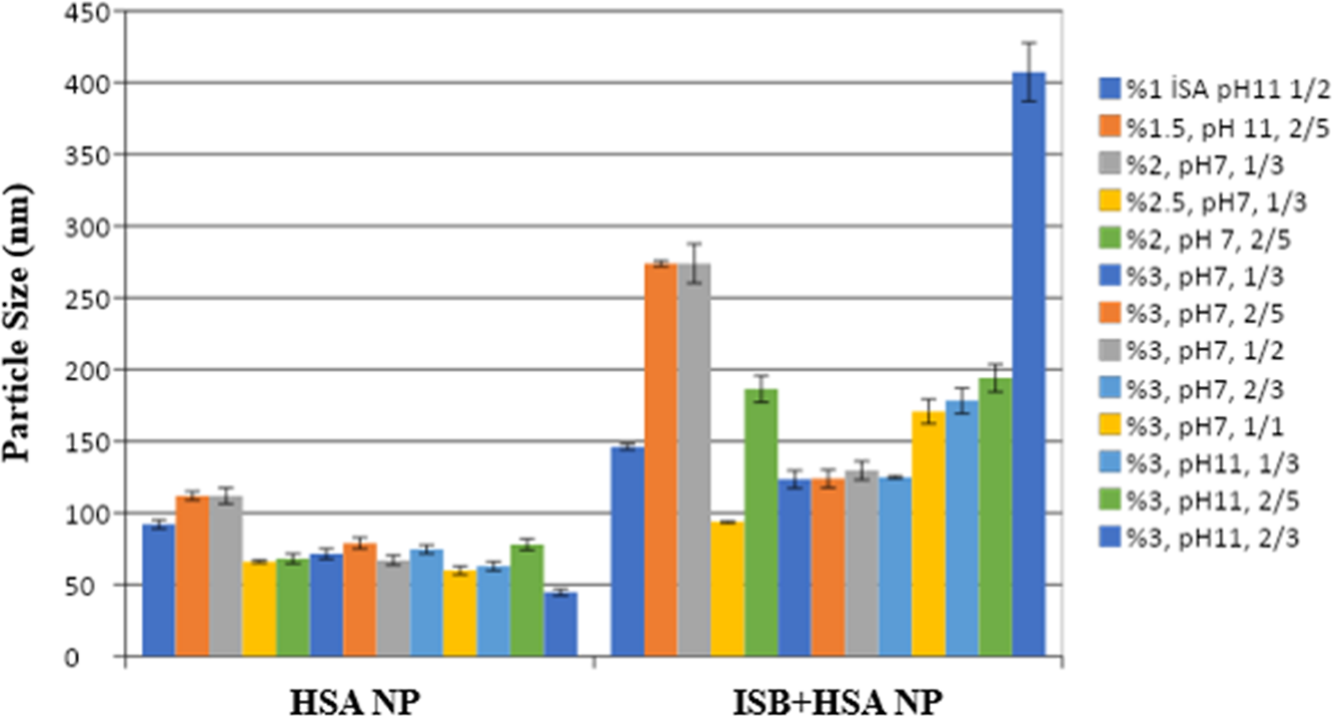
Particle sizes of the formulation were prepared in the absence and presence of ISB.

When the study results were evaluated, it was determined that adding the dye significantly increased the particle size in all formulations (*p* < 0.05).

### Cross-Linker Effect

To examine the effect of the cross-linker on particle size, characterization studies of the formulations prepared determined particle size, zeta potential, and PDI values. Figure 4 shows the formulation’s particle size change before and after the cross-linker addition.

**Figure 4:**
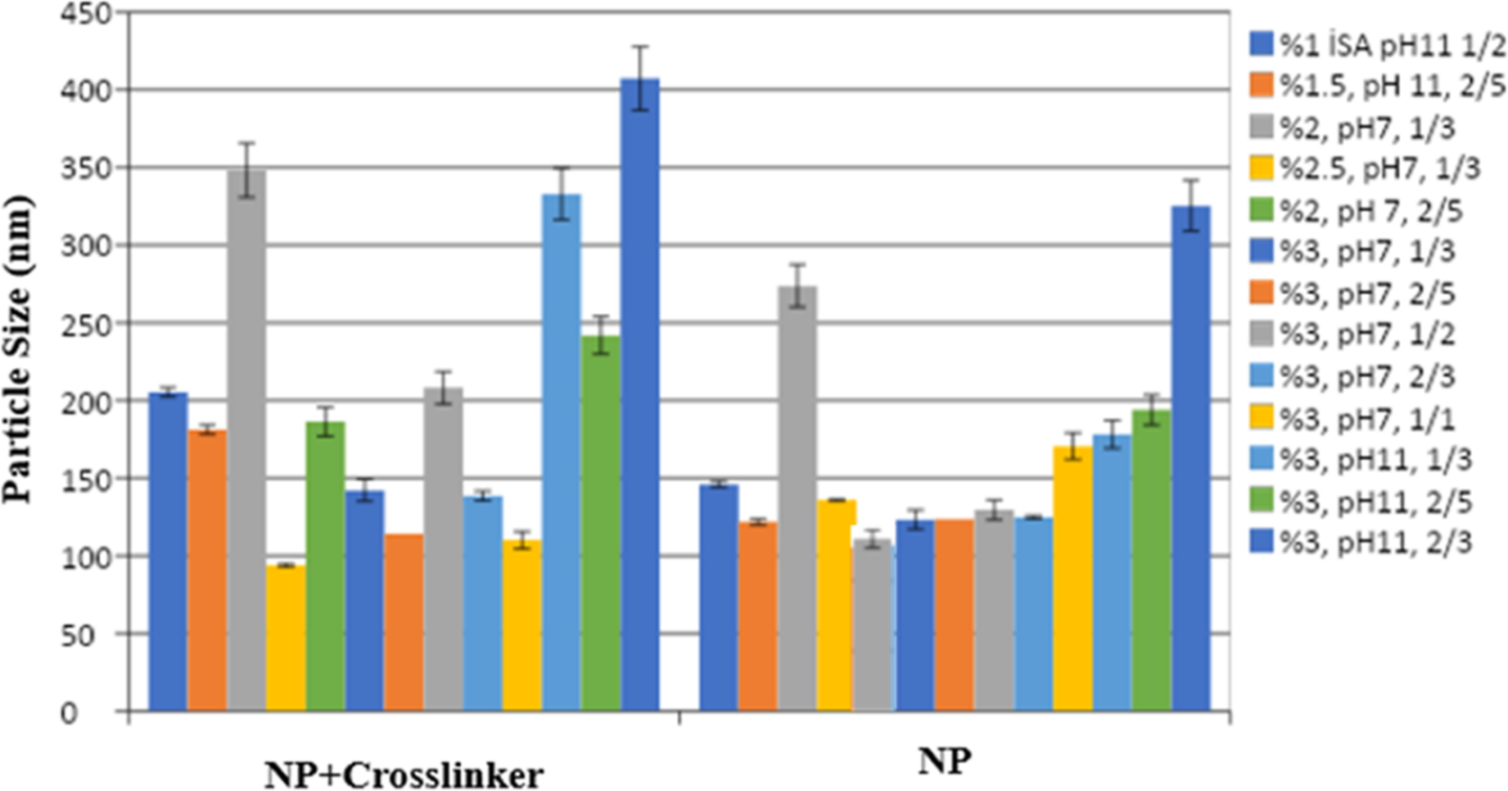
Particle size of formulations before and after the cross-linker.

The effect of the cross-linker on the physicochemical properties of the formulations indicated in Table 2 was investigated. As seen in Figure 4, the particle size of each formulation changed significantly (*p* < 0.05) with the addition of an 8% cross-linker, determined as a result of literature-based preformulation studies. In most formulations, it was found that the particle size decreased after the addition of the cross-linker.

**Table 2:** Contents of the Formulations That Have the Ideal Results in Terms of Particle Size, PDI Value, and Zeta Potential Values

formulation	ISB (%)	HSA (%)	pH	solvent/nonsolvent volume	glutaraldehyde (0.235 μL.mg^–1^ HSA)
F5	1	2.5	7.0	1/3	29.18 μL
F7	1	3.0	7.0	2/5	35.01 μL
F10	1	3.0	7.0	1/1	35.01 μL

The studies determined that the F5, F7, and F10 formulations containing cross-linkers had the most ideal results. Further studies were continued with formulations with ideal properties, whose contents are detailed in Table 2.

### Preparation Colloid of HSA Nanoparticles

The effect of different surfactants on the formulation’s physicochemical properties was investigated during the preparation of nanocolloidal formulations. The particle sizes of ideal formulations prepared with the addition of 1–3% surfactant [Span 20 (S20), Span 80 (S80), Tween 20 (T20), Tween 80 (T80), Cremophor EL (CR), and Colliphor EL (CL)] change were analyzed comparatively. The results are shown in Figure 5.

**Figure 5:**
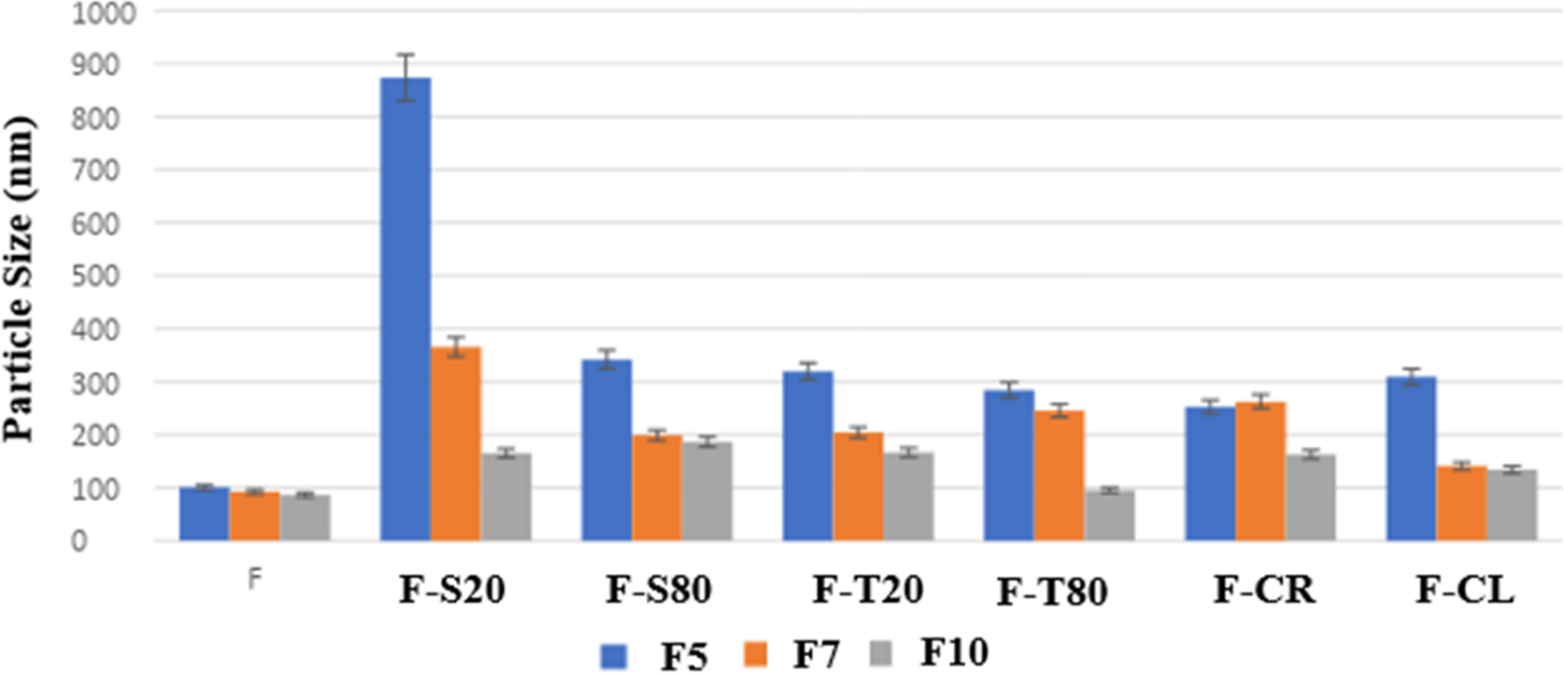
Particle size of formulations prepared using different surfactants.

Studies with F5 and F7 formulations determined that the addition of the surfactant had a significant negative effect on the system’s stability and particle size, even at very low concentrations. Since the particle sizes of the formulations prepared with the addition of the surfactant were far above the target (100 nm), they were excluded from the study. The variation in the particle sizes of the prepared formulations is shown in Figure 5.

The particle size of the F10 formulation prepared with the addition of Tween 80 was found to be within the targeted size range. Therefore, further studies with this formulation were decided to continue.

### Characterization Studies

The particle size, polydispersity index, and zeta potential results of ideal formulations are listed in Table 3.

**Table 3:** Particle Size, Polydispersity Index, and Zeta Potential Results of Formulations

formulation	particle size (nm ± ss)	PDI	zeta potential (mV ± ss)
F5	93.880 ± 1.86	0.24 ± 0.03	–57.4 ± 3.86
F7	113.90 ± 1.91	0.19 ± 0.01	–26.0 ± 2.87
F10	110.00 ± 1.53	0.44 ± 0.02	–50.5 ± 3.79

As indicated in Table 3, the particle sizes of all three formulations were within the target ranges (93.88 ± 1.86–113.90 ± 1.91 nm). In addition, the PDI values of the formulations were below 0.5; that is, the formulations had a homogeneous particle size distribution. All formulations exhibited high colloidal stability, with zeta potential values ranging from −26.0 ± 2.87 mV to −57.4 ± 3.86 mV. According to the results obtained, it was determined that all formulations were suitable for SLN imaging.

### SEM Analysis

As a result of the morphological analysis studies, Figure 6a–c shows the electron micrographs of the SEM analysis of the ideal formulations.

**Figure 6:**
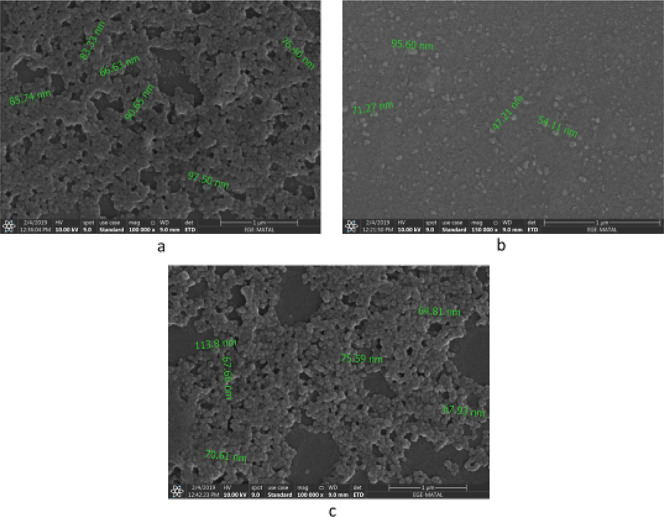
SEM images of ideal formulations F5 (a), F7 (b), and F10 (c).

As seen in the images, all formulations have a spherical shape and a smooth surface. In addition, the particle size results in the images of the formulations obtained by SEM were compatible with the measurement results made with a Zeta Sizer. The particle size results obtained by the Malvern Zeta Sizer and SEM imaging analyses are given in Table 4.

**Table 4:** Particle Size Results Obtained by the Malvern Zeta Sizer and SEM Imaging

formulation	particle size (nm ± ss) (Malvern zeta sizer)	particle size (nm ± ss) (SEM)
F5	93.880 ± 1.86	83.38 ± 10.84
F7	113.90 ± 1.91	67.05 ± 21.56
F10	110.00 ± 1.53	76.73 ± 18.52

### ISB Amount in the Formulations

Table 5 shows the percentages of ISB in the formulations prepared by using the nanoprecipitation method.

**Table 5:** ISB Percentages of Formulations

formulation	ISB (%)
F5	19.74 ± 1.50
F7	15.43 ± 1.62
F10	17.93 ± 3.12

When the results given in Table 5 were examined, it was determined that the amounts of ISB in all three formulations were very close, with ISB in the range of 15–20%.

Different modification studies were carried out to increase the amount of ISB in the formulations. Still, it was determined that the particle size increased significantly with an increase in the amount of ISB in each method. For this reason, it was decided to continue the formulation preparation studies with the ratios used above.

### Stability Studies

The stability of the developed formulations with ideal properties was investigated. Tables 6 and 7 present the stability results of the formulations in the first and third months.

**Table 6:** 1 Month Stability Results of HSA Nanoparticles

	1 month values								
	5 °C			25 °C			40 °C		
for	mean particle size nm ± SD*	PDI* ±SD*	zeta potential (mV*) ± SD*	mean particle size nm ± SD*	PDI* ±SD*	zeta potential (mV*) ± SD*	mean particle size nm ± SD*	PDI* ±SD*	zeta potential (mV*) ± SD*
**F5**	97.95 ± 1.55	0.08 ± 0.01	–45.0 ± 5.2	96.78 ± 0.34	0.09 ± 0.01	–27.7 ± 2.6	112.0 ± 1.80	0.08 ± 0.03	–21.0 ± 2.4
**F7**	85.71 ± 0.49	0.09 ± 0.05	–35.0 ± 3.6	84.52 ± 0.73	0.05 ± 0.02	–26.1 ± 3.4	85.10 ± 0.87	0.06 ± 0.04	–30.9 ± 3.5
**F10**	78.94 ± 1.40	0.39 ± 0.03	–17.9 ± 2.5	86.65 ± 5.27	0.22 ± 0.01	–28.0 ± 1.1	83.95 ± 2.78	0.23 ± 0.02	–23.6 ± 1.4
**F10-T80**	95.99 ± 1.32	0.28 ± 0.01	–21.7 ± 3.8	102.2 ± 2.21	0.37 ± 0.03	–10.9 ± 1.6	93.64 ± 1.63	0.31 ± 0.03	–15.3 ± 1.0

**Table 7:** 3 Month Stability Results of HSA Nanoparticles

	3 months values								
	5 °C			25 °C			40 °C		
for	mean particle size nm ± SD*	PDI* ±SD*	zeta potential (mV*) ± SD*	mean particle size nm ± SD*	PDI* ±SD*	zeta potential (mV*) ± SD*	mean particle size nm ± SD*	PDI* ±SD*	zeta potential (mV*) ± SD*
**F5**	102.2 ± 0.79	0.12 ± 0.01	–18.2 ± 1.6	104.0 ± 1.5	0.11 ± 0.07	–18.2 ± 0.31	105.9 ± 1.97	0.24 ± 0.05	–16.9 ± 0.8
**F7**	83.89 ± 0.12	0.14 ± 0.01	–12.3 ± 0.6	83.9 ± 0.75	0.05 ± 0.01	–17.9 ± 3.1	88.41 ± 0.78	0.13 ± 0.04	–29.7 ± 5.3
**F10**	73.7 ± 1.55	0.35 ± 0.03	–18.9 ± 1.5	77.0 ± 2.85	0.35 ± 0.03	–20.8 ± 1.1	78.64 ± 1.69	0.36 ± 0.01	–25.6 ± 0.7
**F10–T80**	100.5 ± 1.60	0.32 ± 0.02	–27.3 ± 1.1	103.3 ± 1.65	0.09 ± 0.01	–15.0 ± 0.3	99.91 ± 1.05	0.20 ± 0.02	–19.6 ± 3.8

The studies determined that the formulations’ physicochemical properties did not significantly change and were stable.

## Conclusion

HSA-NPs are excellent drug delivery systems that can carry a variety of drugs. The present study carried out a detailed preformulation study to prepare HSA nanoparticles. Various parameters influence the particle size and physical stability of the HSA nanoparticles. The effects of factors such as solvent/nonsolvent ratio, pH, cross-linker, dye, and surfactant concentration on the physicochemical parameters of the formulations were investigated. As a result of the studies, blue dye-loaded HSA nanoparticles in stable nanocolloidal structures with ideal properties were prepared. The optimized formulations exhibited spherical morphology, narrow size distribution (93.88–113.90 nm), low polydispersity indices (PdI <0.5), and high negative zeta potentials (−26.0 to −57.4 mV), ensuring colloidal stability and suitability for passive tumor targeting via the EPR effect. Key findings demonstrated that cross-linking with glutaraldehyde significantly reduced particle size and enhanced stability, while surfactant screening revealed Tween 80 as a viable option for maintaining nanocolloidal integrity. An ISB loading efficiency of 15–20% was achieved without compromising nanoparticle characteristics. Stability studies over 3 months under varied storage conditions (5 °C, 25 °C, and 40 °C) confirmed the robustness of the formulations, with minimal changes in particle size, PdI, and zeta potential. These results highlight the potential of ISB-loaded HSA nanoparticles as a stable, effective alternative to conventional SLN tracers, offering prolonged circulation and reduced degradation.

## Materials and Methods

### Materials

Human Serum Albumin (HSA, purity 96%–99%) and 25% glutaraldehyde solution are from Sigma-Aldrich (USA). All other chemicals are used as received.

### Synthesis of HSA Nanocolloids

The synthesis of HSA nanocolloids was conducted using the nanoprecipitation method (18). The formulation was optimized by changing various parameters like solvent/nonsolvent ratio, pH, cross-linker concentration, etc. HSA was dissolved in deionized water at three different concentrations (1–3%), and the pH of the stock solution was adjusted to pH 7.0. The solutions taken in 6 different beakers with a volume of 5 mL were mixed in a magnetic stirrer at 550 rpm, and other volumes of nonsolvent agent (ethanol) were dropped in at a speed of 1 mL min^–1^. After mixing, the formulation was transferred to a 10–12 Kilodalton dialysis membrane for purification. After dialysis in a water bath at 4 °C for 24 h, solvent, cross-linker, and HSA-free dye were removed from the formulation (18). Characterization studies of the formulations prepared at the end of the dialysis process were performed. Each experimental variable was run in triplicate.

### Nonsolvent Volume Effect

As shown in Figure 7, different ratios (1/3, 2/5, 1/2, 2/3, 1/1, and 1/0.5) of the nonsolvent (ethanol) were added to the stock solutions of HSA at pH 7.0 (1–3%) using 10 mM PBS. The effect of nonsolvent volume was investigated by performing characterization studies of the prepared formulations.

**Figure 7:**
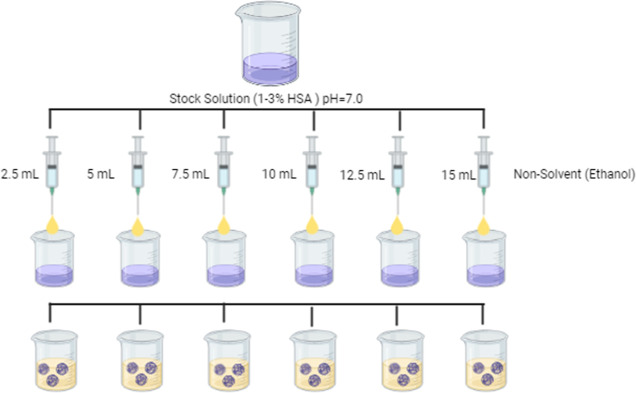
Effect of nonsolvent volume on particle size based on 1–3% of the stock solution adjusted to pH 7.0. Created with BioRender.com.

### pH Effect

HSA nanoparticles were prepared at pH 7 and 11, and the effect of pH on formulations was investigated.

### ISB Concentration Effect

The dye solution (1 mg mL^–1^) was added to the HSA stock solutions (1–3%), whose pH was adjusted to 7 and 11. Different volumes of the nonsolvent were added for nanoprecipitation, and after the mixing and dialyzing process, the physicochemical properties of the prepared formulations were examined. The preparation method is schematized in Figure 8.

**Figure 8:**
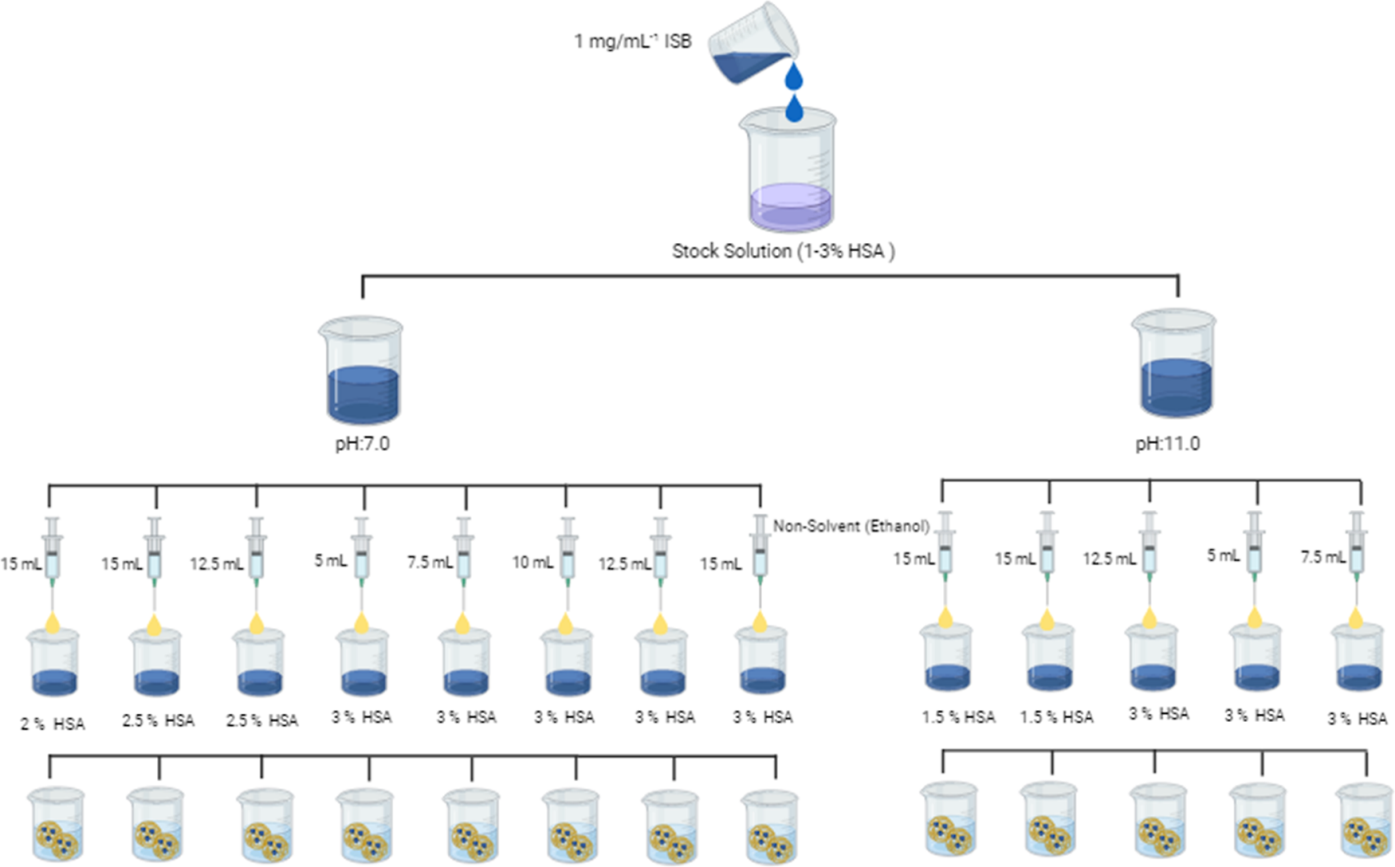
Effect of ISB concentration.

### Cross-Linker Effect

Formulations were prepared as shown in Figure 8. After the addition of the nonsolvent, 8% glutaraldehyde (0.235 μL mg^–1^ HSA) was added as a cross-linker. The physicochemical properties of the formulations were examined after mixing and purification by dialysis.

### Preparation Colloid of HSA Nanoparticles

The effect of using different surfactants on the physicochemical properties of the formulation was investigated during the preparation of the nanocolloidal formulation. The formulations found to have the closest ideal properties due to the preformulation studies were prepared with the addition of S20, S80, T20, T80, CR, and CL (1%).

### Characterization Studies

#### Particle Size, PDI, and Zeta Potential

The prepared formulations were evaluated in terms of aggregate formation, particle size, and polydispersity index with a Malvern Zeta Sizer (Nano-ZS) in the particle size range of 3–1000 nm at room temperature, with an angle of 173°. The zeta potentials of the formulations were determined with Malvern Zeta Sizer (Nano-ZS) at a field strength of 40 V cm^–1^ at 25 °C, the dielectric constant of 78.5, and the conductivity of 5 mS cm^–1^, using DTS 1060C zeta cuvette. Samples were diluted with filtered, bidistilled water before evaluation.

### SEM Analysis

The size and surface properties of the formulations were examined under the Philips’s brand XL 30S FEG model SEM. For this purpose, the samples were coated with gold–palladium on an aluminum grid, and scanning was carried out on the coated samples at a magnification range of ×12.000 and under 4 kV incremental voltage conditions.

### ISB Amount in Formulations

Formulations with appropriate particle size, PDI value, and zeta potential prepared by using the nanoprecipitation method were selected, and the contained ISB amount was determined. The study combined dialysis and sonication processes, and the % ISB content was calculated using the formulation described below.

1 %ISB=[(A−B)/C]×100

*A*: amount of ISB added to the formulation *B*: amount of ISB detected in the dialysis medium *C*: amount of ISB added to the formulation.

### Stability Studies

Studies to examine the stability of the developed formulation were carried out at 5 ± 3 °C (refrigerated) and 25 ± 5 °C, 60 ± 5% relative humidity, and 40 ± 5 °C, 75 ± 5% relative humidity by the stability guideline. In the stability study, the samples were checked for 3 months at *t* = 0, that is, at the time of onset, at the first and third months for physical appearance, particle size, polydispersity index, and zeta potential.

### Statistical Analysis

Data are expressed as the mean ± SD of at least three experiments. They were statistically obtained by variance analysis, while one-way ANOVA compared the means at a significant level 0.05. All calculations were performed using GraphPad Prism 6.0 statistical software (GraphPad Software, CA, USA).

## Abbreviations

CR: Cremophor EL
CL: Colliphor EL
DLS: dynamic light scattering
EPR: enhanced permeability and retention effect
HSA: human serum albumin
ISB: isosulfan blue
S20: span 20
SEM: scanning electron microscope
S80: span 80
T20: tween 20
T80: tween 80.
